# Tang-Luo-Ning, a Traditional Chinese Medicine, Inhibits Endoplasmic Reticulum Stress-Induced Apoptosis of Schwann Cells under High Glucose Environment

**DOI:** 10.1155/2017/5193548

**Published:** 2017-11-21

**Authors:** Weijie Yao, Xinwei Yang, Jiayue Zhu, Biane Gao, Renhui Liu, Liping Xu

**Affiliations:** Beijing Key Lab of TCM Collateral Disease Theory Research, School of Traditional Chinese Medicine, Capital Medical University, Beijing, China

## Abstract

Tang-Luo-Ning (TLN) has a definite effect in the clinical treatment of diabetic peripheral neuropathy (DPN). Schwann cells (SCs) apoptosis induced by endoplasmic reticulum stress (ER stress) is one of the main pathogeneses of DPN. This study investigates whether TLN can inhibit SCs apoptosis by inhibiting ER stress-induced apoptosis. Our previous researches have demonstrated that TLN could increase the expression of ER stress marker protein GRP78 and inhibited the expression of apoptosis marker protein CHOP in ER stress. In this study, the results showed that TLN attenuated apoptosis by decreasing Ca^2+^ level in SCs and maintaining ER morphology. TLN could decrease downstream proteins of CHOP including GADD34 and Ero1*α*, while it increased P-eIF2*α* and decreased the upstream proteins of CHOP including P-IRE1*α*/IRE1*α* and XBP-1, thereby reducing ER stress-induced apoptosis.

## 1. Background

Diabetic peripheral neuropathy (DPN) is a major complication of diabetes [1] and its pathogenesis is complex. Schwann cells (SCs) apoptosis induced by hyperglycemia is involved in the pathogenesis of DPN [2]. SCs, as the peripheral myelin-forming cells, play an important role in maintaining the structure and function of peripheral nerves [3]. SCs apoptosis induced by hyperglycemia is a key factor in decreasing nerve conduction velocity and increasing thermal perception threshold, axon atrophy, and demyelination of DPN [4, 5].

Recent studies have shown that endoplasmic reticulum stress- (ER stress-) induced apoptosis is involved in the pathogenesis of DPN [6, 7]. ER stress results from the accumulation of unfolded proteins or misfolded proteins in the ER. Unfolded protein response (UPR) can be activated for restoring homeostasis of ER. But persistent ER stress can also induce apoptosis [8]. Therefore, we explore the mechanism of SCs apoptosis induced by ER stress and provide new ideas for further studying the pathogenesis of DPN.

Tang-Luo-Ning (TLN) is a traditional Chinese medicine (TCM), designed based on Huangqi Guizhi Wuwu decoction, and the treatments were replenishing Qi and nourishing Yin, nourishing liver and kidney, eliminating blood stasis, and dredging collaterals under the guidance of collateral disease theory. Previous clinical studies confirmed that TLN could improve the pain and numbness of DPN patients, decrease diabetic neuropathy score, and improve the sensory and motor nerve conduction velocity, thus improving the patient's neurological function. The total effective rate can be up to 93.8% [9, 10]. Experimental studies also showed that improving DPN is associated with antioxidant stress and decreasing SCs apoptosis [11]. Oxidative stress is closely related to ER stress. They interplay each other and both aggravate SCs apoptosis [12]. Our previous study also demonstrated that TLN could increase the expression of ER stress marker protein GRP78 and inhibited the expression of apoptosis marker protein CHOP in ER stress and then increase the expression of Bcl-2 and decrease the expression of Bax [11]. This study aims to investigate whether TLN can inhibit CHOP-related pathway to inhibit ER stress-induced apoptosis.

## 2. Materials and Methods

### 2.1. Preparation of TLN

TLN is composed of 15 g of Huangqi, 15 g of Danshen, 15 g of Gouji, 12 g of Chuanniuxi, 10 g of Yanhusuo, 15 g of Mugua, 12 g of Chishao, and 15 g of Jixueteng. The specimen of 8 crude drug materials has been stored in a publicly available herbarium in the School of Traditional Chinese Medicine. The mixture was decocted twice; then filtrate was evaporated to TLN powder. The quality control method of TLN was previously described by Yang et al. [13]. TLN powder was dissolved in distilled water. Then, TLN solution was used for intragastric administration.

### 2.2. Preparation of TLN Serum

30 SPF-grade male Sprague-Dawley (SD) rats (200 ± 20 g) were obtained from Experimental Animal Center of Capital Medical University. All experimental procedures were conducted in accordance with the protocol for using animals, which were approved by the Ethics Review Committee for Animal Experimentation of Capital Medical University (Ethical Inspection Number: AEEI-2014-086). Rats were maintained at room temperature (20–25°C) under constant humidity (40–70%) under a 12-hour light-dark cycle in a pathogen-free laboratory and allowed free access to water and standard laboratory diet. Rats were randomly divided into three groups, 15 for control, 10 for trimethylamine N-oxide (TMAO, Sigma), and 10 for TLN groups, and were intragastrically given distilled water, 110 mg/kg/day TMAO suspension, and 10.9 g crude drug/kg/day TLN, respectively, for 8 days. One hour after the last administration, rats were anesthetized with 10% chloral hydrate (i.p., 0.35 g/kg body weight). Blood was sterilely collected through the ventral aorta. After settling for 2 hours at room temperature, the blood samples were centrifuged at 3000 r/min at 4°C for 15 min and inactivated at 56°C for 30 min. The samples were stored at −80°C after being filtered through a microfiltration membrane (0.22 *μ*m).

### 2.3. Cell Culture

RSC96 cells (obtained from the American Type Culture Collection, ATCC) were cultured in Dulbecco's Modified Eagle's Medium (DMEM) modified to contain 4 mmol L-glutamine, 25 mmol/L glucose, 1 mmol sodium pyruvate, 1500 mg/L sodium bicarbonate, and 10% fetal bovine serum (FBS, Gibco/Invitrogen Corporation, Carlsbad, CA, USA) in a humidified atmosphere of 5% CO_2_ at 37°C.

### 2.4. Measurement of Cell Viability

RSC96 cells were seeded at a suitable density (4 × 10^3^ cells/well for 24 hours and 3 × 10^3^ cells/well for 48 hours) in 96-well plate and allowed to attach overnight and then were treated with 150 mM glucose (Sigma) and various concentrations of TLN serum (10%, 1%, and 0.1%) for 24 hours and 48 hours. After treatment, 100 *μ*L of MTT (5 mg/mL, Sigma) solution was added and incubated for 4 hours at 37°C. Then MTT solution was removed and DMSO was added (150 *μ*L/well) for incubation for 10 min at room temperature. Cell viability was measured at 490 nm by using SpectraMax Plus 384 Microplate Reader (Molecular Devices, Sunnyvale, CA, USA). The results were indicated as a percentage of the absorbance of 25 mM glucose cells.

### 2.5. Measurement of Ca^2+^ Level

RSC96 cells were seeded at a suitable density (4 × 10^5^ cells/well for 24 hours and 3 × 10^5^ cells/well for 48 hours) in 6-well plate and allowed to attach overnight and then were treated differently as described above in Section 2.4. After that, RSC96 cells were lysed by 0.25% trypsin (without EDTA) and collected, incubated by Fluo-3 (0.5 *μ*M, Beyotime) in 37°C for 30 min keep in dark place, and then analyzed by BD LSRFortessa™ flow cytometry (BD Biosciences, San Jose, CA, USA).

### 2.6. Ultrastructure Observation of ER

RSC96 cells were seeded at a suitable density (4 × 10^5^ cells/well for 24 hours and 3 × 10^5^ cells/well for 48 hours) in 6-well plate and allowed to attach overnight and then were treated differently as described above in Section 2.4. After that, RSC96 cells were lysed with 0.25% trypsin (without EDTA) and brought together by centrifuging (2000 rpm × 5 min) and then were fixed with 2.5% glutaraldehyde for 3 hours followed by storage in phosphate buffer (PB) and then were sent to the Electron Microscopy Center of Capital Medical University for ultrastructure observation.

### 2.7. High Content Analysis

RSC96 cells were seeded at a suitable density (4 × 10^3^ cells/well for 24 hours and 3 × 10^3^ cells/well for 48 hours) in 96-well plate and allowed to attach overnight and then were treated differently as described above in Section 2.4. After that, RSC96 cells were fixed with 4% paraformaldehyde at room temperature for 30 min and then permeabilized with 0.5% Triton-X100 in ice bath for 30 min. After being blocked by 3% BSA at room temperature for 30 min, RSC96 cells were incubated with anti-GADD34 (1 : 50, Santa Cruz, sc-8327) and anti-XBP-1 (1 : 200, Abcam, ab37152) antibodies at 4°C overnight, respectively. RSC96 cells were incubated with goat anti-rabbit IgG FITC (1 : 100) at room temperature for 2 hours (in the dark) and then were counterstained with DAPI (Roche) at room temperature for 5 min. Cell images were acquired by using Thermo Fisher Scientific Cellomics ArrayScan VTI High Content Screening Reader and images were analyzed by using the Compartmental Analysis BioApplication (Thermo Fisher Scientific). The results were indicated as a percentage of the average intensity of 25 mM glucose cells.

### 2.8. Western Blot Analysis

RSC96 cells were seeded at a suitable density (4 × 10^5^ cells/well for 24 hours and 3 × 10^5^ cells/well for 48 hours) in 6-well plate and allowed to attach overnight and then were treated differently as described above in Section 2.4. After that, cells were lysed in RIPA lysis buffer (including proteinase inhibitor cocktail and phosphatase inhibitor cocktail) in ice bath for 15 min and centrifuged (12,000 rpm/min, 4°C) for 10 min; then supernatant was collected. Then concentration of protein was measured by BCA Protein Assay Kit and DTT loading buffer was added to prepare samples. Equal amounts of protein (20 *μ*g) were separated by electrophoresis in 10% SDS-PAGE gel and transferred to a 0.45 *μ*m PVDF membrane (Millipore) by wet transfer system (Bio-Rad, USA) or semidry transfer system (Wealtec, USA). Blots in PVDF membrane were blocked with 5% nonfat-dried milk (phosphoproteins were blocked with 2% bovine serum albumin) for 2 hours at room temperature and then incubated with primary antibodies overnight at 4°C. Then goat anti-mouse and goat anti-rabbit antibodies were added to corresponding blots and reacted for 1 hour at room temperature followed by incubation in electrochemiluminescence (ECL) reagent (Millipore). Blots were exposed to X-film to form image. *β*-Actin antibody (1 : 2000, Zhongshan Golden Bridge, TA-09) was to ensure normalization of results. Quantitative analysis was measured by using ImageJ software. The following primary antibodies were used: mouse monoclonal anti-Ero1*α* (1 : 500, Santa Cruz, sc-100805); rabbit polyclonal anti-IRE1*α* (1 : 2000, Santa Cruz, sc-20790), anti-P-IRE1*α* (1 : 2000, Abcam, ab48187), and anti-P-eIF2*α* (1 : 1500, Santa Cruz, sc-293100).

### 2.9. Statistical Analysis

SPSS17.0 was used to analysis data. Data was presented as mean ± standard error of mean (SEM). Between-group differences were assessed using one-way analysis of variance (ANOVA). *P* < 0.05 was considered to be significant.

## 3. Results

### 3.1. TLN Inhibited High Glucose-Induced Cytotoxicity of RSC96 Cells

**Figure 1:**
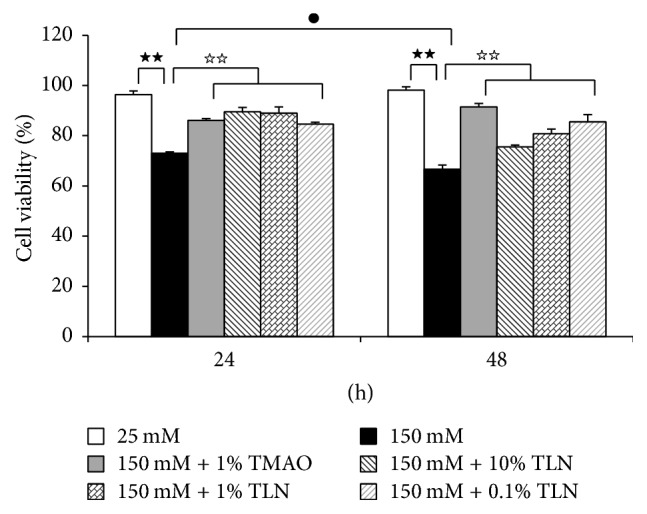
*TLN inhibited high glucose-induced cytotoxicity of RSC96 cells.* Data were analyzed by one-way ANOVA followed by least significant difference. Data were shown as mean ± SEM (*n* = 4). ^★★^ *P* < 0.01 versus 25 mM glucose; ^☆☆^ *P* < 0.01 versus 150 mM glucose. ^●^ *P* < 0.05, 150 mM glucose at 24 hours versus 150 mM glucose at 48 hours.

Cell viability test was conducted by MTT assay and it aimed to evaluate the protective effect of TLN serum for SCs. The results showed that, after incubation with high glucose for 24 hours and 48 hours, cell viability in 150 mM glucose group decreased significantly (*P* < 0.01); TLN serum could increase RSC96 cell viability in high glucose environment (*P* < 0.01) (Figure 1).

### 3.2. TLN Decreased High Glucose-Induced Ca^2+^ Level of RSC96 Cell

**Figure 2:**
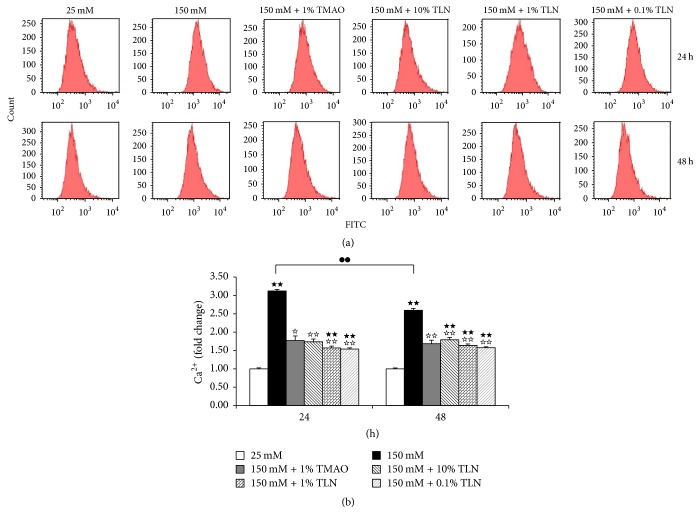
*TLN decreased high glucose-induced Ca* ^2+^ *level of RSC96 cell.* (a) Image of Ca^2+^ level in RSC96 cells measured by flow cytometry. (b) Summarized data of Ca^2+^, normalized as fold change of 25 mM glucose. Data were analyzed by one-way ANOVA followed by least significant difference. Data were shown as mean ± SEM (*n* = 4). ^★★^ *P* < 0.01 versus 25 mM glucose; ^☆☆^ *P* < 0.01 and ^☆^ *P* < 0.05 versus 150 mM glucose; ^●●^ *P* < 0.01 150 mM glucose at 24 hours versus 150 mM glucose at 48 hours.

Increasing Ca^2+^ level is recognized to be a factor for cell apoptosis [14, 15]. Results showed that Ca^2+^ level in 150 mM glucose group increased significantly compared with 25 mM group (*P* < 0.01), and TLN serum decreased Ca^2+^ level significantly (*P* < 0.01), thus alleviating high glucose-induced apoptosis (Figure 2).

### 3.3. TLN Protected the Integrity of the ER Morphology under High Glucose Environment

**Figure 3:**
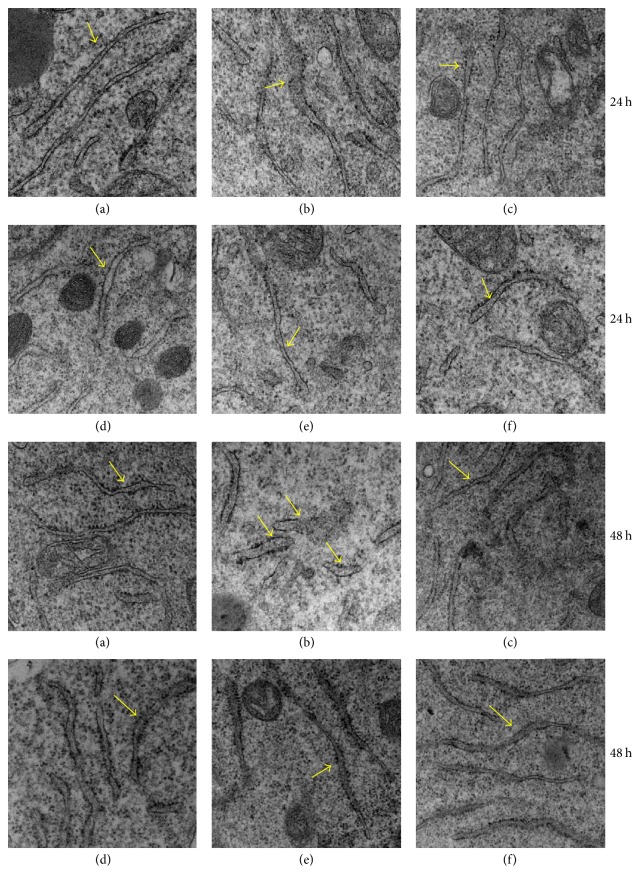
*TLN protected the integrity of the ER morphology under high glucose environment.* Ultramicrostructure of ER (magnification, 8,000x): the arrow indicates the ER morphology. (a)–(f) represent 25 mM glucose, 150 mM glucose, 150 mM glucose + 1% TMAO, 150 mM glucose + 10% TLN, 150 mM glucose + 1% TLN, and 150 mM + 0.1% TLN group, respectively.

As shown in Figure 3, ER membrane structure in 25 mM glucose group is clear and intact (Figure 3(a) of 24 hours and 48 hours), while in 150 mM glucose group morphology of ER partially swelled and was not uniform (Figure 3(b) of 24 hours and 48 hours); particularly in 150 mM glucose group at 48 hours, fragmental morphology of ER appeared (Figure 3(b) at 48 hours). In 150 mM glucose + TLN group, morphology of ER tended to have integral structure (Figures 3(d), 3(e), and 3(f)); it demonstrates that TLN can protect the integrity of the ER morphology.

### 3.4. TLN Regulated Related Protein Expression of ER Stress-Induced Apoptotic Pathway of RSC96 Cells by High Glucose

**Figure 4:**
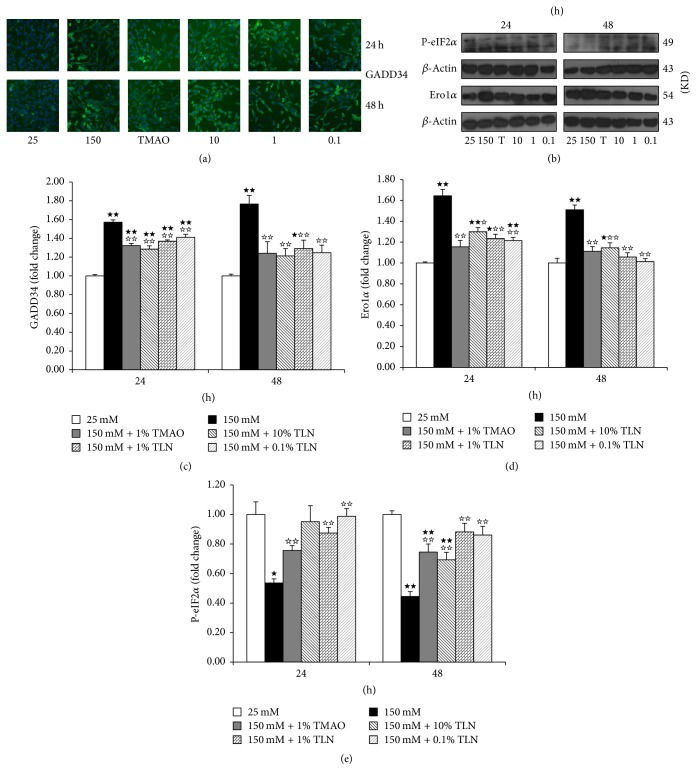
*TLN can decrease the expression of GADD34 and Ero1α and increase the expression of P-eIF2α in high glucose-induced RSC96 cells.* (a) Images of GADD34 relative protein level measured by high content analysis; images were viewed at a magnification of 10x. (b) Images of Ero1*α* and P-eIF2*α* relative protein level measured by Western blot, normalized to *β*-actin. ((c)–(e)) Summarized data of GADD34, Ero1*α*, and P-eIF2*α*, normalized as fold change of 25 mM glucose group. Data were analyzed by one-way ANOVA followed by least significant difference. Data were shown as mean ± SEM (*n* = 4). ^★★^ *P* < 0.01 and ^★^ *P* < 0.05 versus 25 mM glucose; ^☆☆^ *P* < 0.01 and ^☆^ *P* < 0.05 versus 150 mM glucose.

CHOP can induce apoptosis directly or indirectly [16]. Our previous study also demonstrated that TLN could increase the expression of ER stress marker protein GRP78 and inhibited the expression of apoptosis marker protein CHOP in ER stress and then increased the expression of Bcl-2 and decreased the expression of Bax [11]. We further measured the expressions of GADD34, P-eIF2*α*, and Ero1*α* which are CHOP-related proteins. We first found that the expressions of GADD34 and Ero1*α* in 150 mM glucose group increased significantly compared with the control group (*P* < 0.01) (Figures 4(c) and 4(d)); they showed the same trend compared with the expression of CHOP, while P-eIF2*α* was inhibited by GADD34 and decreased (*P* < 0.05 and *P* < 0.01) (Figure 4(e)). TLN serum can decrease the expression of GADD34 and Ero1*α* but increase the expression of P-eIF2*α* (*P* < 0.05 and *P* < 0.01) (Figures 4(c), 4(d), and 4(e)). The results also show that TLN can inhibit ER stress-induced apoptosis.

**Figure 5:**
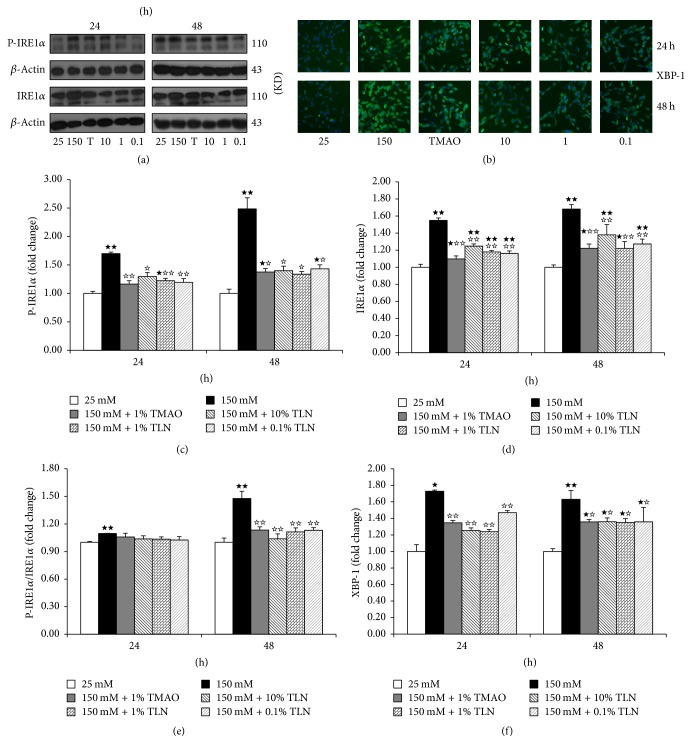
*TLN can decrease the expression of P-IRE1α/IRE1α and XBP-1 in high glucose-induced RSC96 cells.* (a) Images of P-IRE1*α* and IRE1*α* relative protein level measured by Western blot, normalized to *β*-actin. (b) Images of XBP-1 relative protein level measured by high content analysis; images were viewed at a magnification of 10x. ((c)–(f)) Summarized data of P-IRE1*α*, IRE1*α*, P-IRE1*α*/IRE1*α*, and XBP-1, normalized as fold change of 25 mM glucose group. Data were analyzed by one-way ANOVA followed by least significant difference. Data were shown as mean ± SEM (*n* = 4). ^★★^ *P* < 0.01 and ^★^ *P* < 0.05 versus 25 mM glucose; ^☆☆^ *P* < 0.01 and ^☆^ *P* < 0.05 versus 150 mM glucose.

Because TLN decreased ER stress-related apoptotic protein CHOP induced by high glucose, we hypothesized whether it resulted from downregulating the expression of CHOP upstream proteins. IRE1*α* is one of three UPR transmembrane proteins that can induce CHOP protein expression by inducing expression of XBP-1 [17]. Therefore, we continued to measure the expressions of IRE1*α* and XBP-1 to observe whether TLN can inhibit the ER stress-induced apoptosis by inhibiting their expression. We found that the expressions of P-IRE1*α*/IRE1*α* and XBP-1 in 150 mM glucose group increased significantly compared with the 25 mM glucose group (*P* < 0.05 and *P* < 0.01) (Figures 5(c)–5(f)); they showed the same trend compared with the expression of CHOP. The expressions of P-IRE1*α*/IRE1*α* at 48 hours and XBP-1 at both 24 hours and 48 hours decreased significantly in 150 mM glucose + TLN group compared with the 150 mM glucose group (*P* < 0.05 and *P* < 0.01) (Figures 5(c)–5(f)). This demonstrated that TLN can inhibit ER stress-induced apoptosis by inhibiting the expressions of IRE1*α* and XBP-1.

## 4. Discussion

TCM serum has been widely used in the mechanism study of compound of traditional Chinese medicine in vitro. It excludes interference of various factors and is close to the real process of pharmacological effects in Chinese herbal compound [18, 19]. Based on previous DPN rat models, we had verified that TLN can inhibit ER stress-induced apoptosis of sciatic nerve of DPN rats and alleviated DPN [13]. In order to further explore the mechanism of TLN on DPN, in this study, a model of SCs cultured in high glucose environment was used and treated with TLN serum. Previous studies have also found that the main chemical constituents of the TLN such as paeoniflorin and salvianolic acid B can be detected in TLN serum (see Supplementary Material available online at https://doi.org/10.1155/2017/5193548).

SCs apoptosis is one of the main pathogeneses of DPN [2]. Our previous study had demonstrated that TLN could inhibit high glucose-induced apoptosis [11]. ER is the main storage organelle of calcium and intracellular Ca^2+^ is indispensable for the development of ER. Overload calcium is identified to be a factor for cell apoptosis [14, 15] and participates in ER stress [20, 21]. In this study, Ca^2+^ level in 150 mM glucose group increased significantly compared with 25 mM group; it suggested that intracellular environmental homeostasis was destroyed and led to apoptosis. TLN serum decreased Ca^2+^ level significantly; it suggested that TLN could inhibit high glucose-induced apoptosis. We also found that the Ca^2+^ level in the 150 mM glucose group at 48 hours was significantly decreased compared to that in the 150 mM glucose group at 24 hours; we speculate that the reason may be that the cell membrane structure at 48 hours was damaged more seriously compared with that at 24 hours. PI can stain the nucleus through late apoptosis and dead cells but not the complete cell membrane. From our previous research of the Annexin V/PI staining [11], we can find that PI staining increased significantly at 48 hours compared with 24 hours; it suggested that the quantity of late apoptosis and dead cells increased; cell membrane structure was destroyed and led to Ca^2+^ outflow and Ca^2+^ levels decreased; this verified our guess.

The accumulation of unfolded proteins and misfolded proteins in the ER resulted in defect in the ER homeostasis and then induced ER stress [20, 21]. In order to restore homeostasis, UPR, which is involved in three ER-transmembrane transducers, PERK, IRE1*α*, and ATF6, and ER chaperone GRP78 were activated [8]. We first observed the morphology of ER and found that in 150 mM group at 24 hours the morphology of ER partially swelled and in 150 mM group at 48 hours, fragmental morphology of ER appeared; it is the result of protein overaccumulation in the ER and intracellular environmental homeostasis was destroyed and then induced ER stress. In 150 mM glucose + TLN group, morphology of ER tended to have integral structure; it demonstrates that TLN can maintain the integrity of the ER morphology.

When ER stress lasts for long time, ER homeostasis cannot be restored in time; ER apoptosis mechanism will be activated [8]. Our previous study also demonstrated that TLN could increase the expression of ER stress marker protein GRP78 and inhibited the expression of apoptosis marker protein CHOP in ER stress and then increased the expression of Bcl-2 and decreased the expression of Bax [11]. Upregulation of CHOP can also induce the expression of Ero1*α* and GADD34 [22–26], Ero1*α* can trigger apoptosis directly, and GADD34 can further make P-eIF2*α* (function is slowing down or even suspending misfolded proteins synthesis) dephosphorylated and then result in misfolded proteins synthesis conduct. This study showed that TLN serum could decrease the expression of Ero1*α* and GADD34 but increase the expression of P-eIF2*α*; it demonstrates that TLN can inhibit apoptosis and the folding of misfolded proteins.

IRE1*α*, as the transmembrane protein for UPR, plays a critical role in ER stress-induced apoptosis [17]. When ER stress occurs, free IRE1*α* is activated by dimerization and phosphorylation and induces downstream expression of XBP-1s by cleavage of transcription factor XBP-1 mRNA and ultimately activates downstream apoptotic protein CHOP [27]. In this study, we found that TLN serum could decrease the expressions of P-IRE1*α*/IRE1*α* and XBP-1; it demonstrates that TLN can inhibit CHOP-induced apoptosis by inhibiting IRE1*α* phosphorylation followed with inhibiting the cleavage of XBP-1.

In this study, we selected TMAO as a positive control drug. TMAO is a natural chemical chaperone that can attract chaperone proteins to treat protein-folding diseases and eliminate abnormal accumulation of misfolded proteins and thus alleviate ER stress and treat DPN effectively [28, 29]. Studies have shown that it can significantly reduce the expressions of CHOP and P-IRE1*α*/IRE1*α* and improve neurological function [30–32], so we select TMAO as positive control drug.

## 5. Conclusion

**Figure 6:**
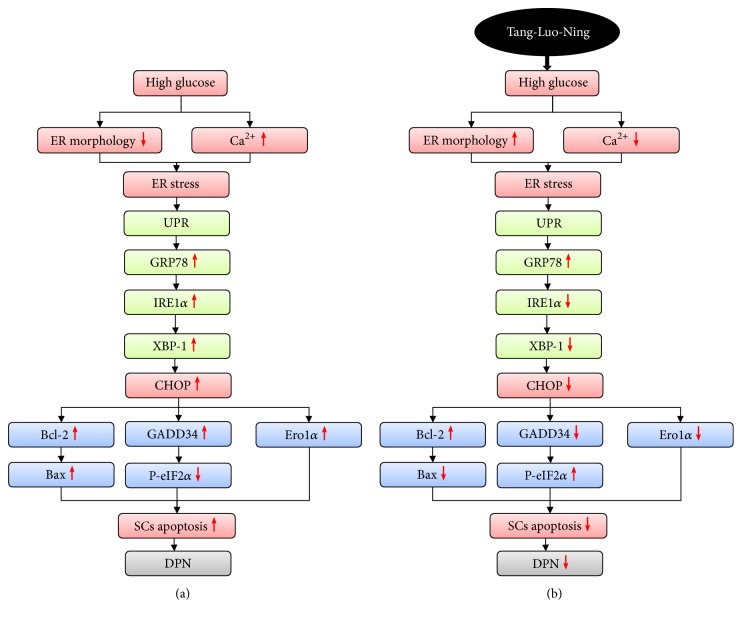
*(a) The mechanism of ER stress-induced apoptosis of SCs under high glucose*. *(b) TLN inhibits high glucose-induced ER stress-related apoptosis of SCs*.

In summary, this study demonstrates that TLN can decrease SCs Ca^2+^ level induced by high glucose. It is related to the inhibition of CHOP-related pathway of ER stress apoptotic pathway. These results suggest a therapeutic pathway for TLN in decreasing SCs apoptosis in DPN (Figure 6).

## Supplementary Material

Figure 1: HPLC-MS/MS total ion chromatogram and extract ion chromatogram in positive ion mode of TLN extract, TLN serum and blank serum. Figure 2: TLN increased the expression of GRP78 and Bcl-2 while decrease the expression of CHOP and Bax in high glucose-induced RSC96 cells.
