# Supplementary material for: Tang-Luo-Ning, a Traditional Chinese Medicine, Inhibits Endoplasmic Reticulum Stress-Induced Apoptosis of Schwann Cells under High Glucose Environment
Source: Evid Based Complement Alternat Med. 2017 Nov 21;2017:5193548. doi: 10.1155/2017/5193548 (PMC5736904; doi:10.1155/2017/5193548)
Supplement: Supplementary file 1 — Figure 1: HPLC-MS/MS total ion chromatogram and extract ion chromatogram in positive ion mode of TLN extract, TLN serum and blank serum. Figure 2: TLN increased the expression of GRP78 and Bcl-2 while decrease the expression of CHOP and Bax in high glucose-induced RSC96 cells. [file 5193548.f1.pdf]

1.1 Identification method of the main chemical constituents in TLN serum

1ml rat serum sample was mixed with 2 ml of acetonitrile evenly, after centrifuged for 10 min (12000 g, 4 ℃), the supernatant was filtered with a 0.22 μm microporous membrane. The solution was analyzed using HPLC-MS/MS. The LC-MS system was equipped with Thermo Accela UHPLC liquid chromatography system and Thermo Fisher LTQ-Orbitrap mass spectrometer detector. An agilent poroshell 120 EC-C18 reversed-phase column (4.6 mm × 150 mm, 2.7 μm) was used. The mobile phase consisted of water with 0.1% formic acid (phase A) and acetonitrile (phase B). Gradient elution program was as follow: 0-2 min，1% B；2-5 min，1-20% B；5-15 min，20-75% B； 15-16 min，75-95% B； 16-24 min，95% B； 24-25 min，95-1% B； 25-32 min，1% B. The flow rate was 0.3 mL/min. The column temperature was 30 ℃. A 10 μL sample was injected into HPLC by auto-sampler. The parameters were as follow: ESI ion source; positive ion detection mode; capillary temperature, 350 ℃; cone hole voltage, 5.0 kV; sheath gas, auxiliary gas and purge gas were 40 arb, 5 arb and 0 arb; cracking energy 35%.

Identification results of the chemical constituents in the TLN serum

TLN extract, TLN serum and Blank serum were analyzed, the TLN serum contains **chlorogenic acid**, **paeoniflorin** and **salvianolic acid B** through the analysis of total ion chromatogram and extract ion chromatogram (Fig.1).

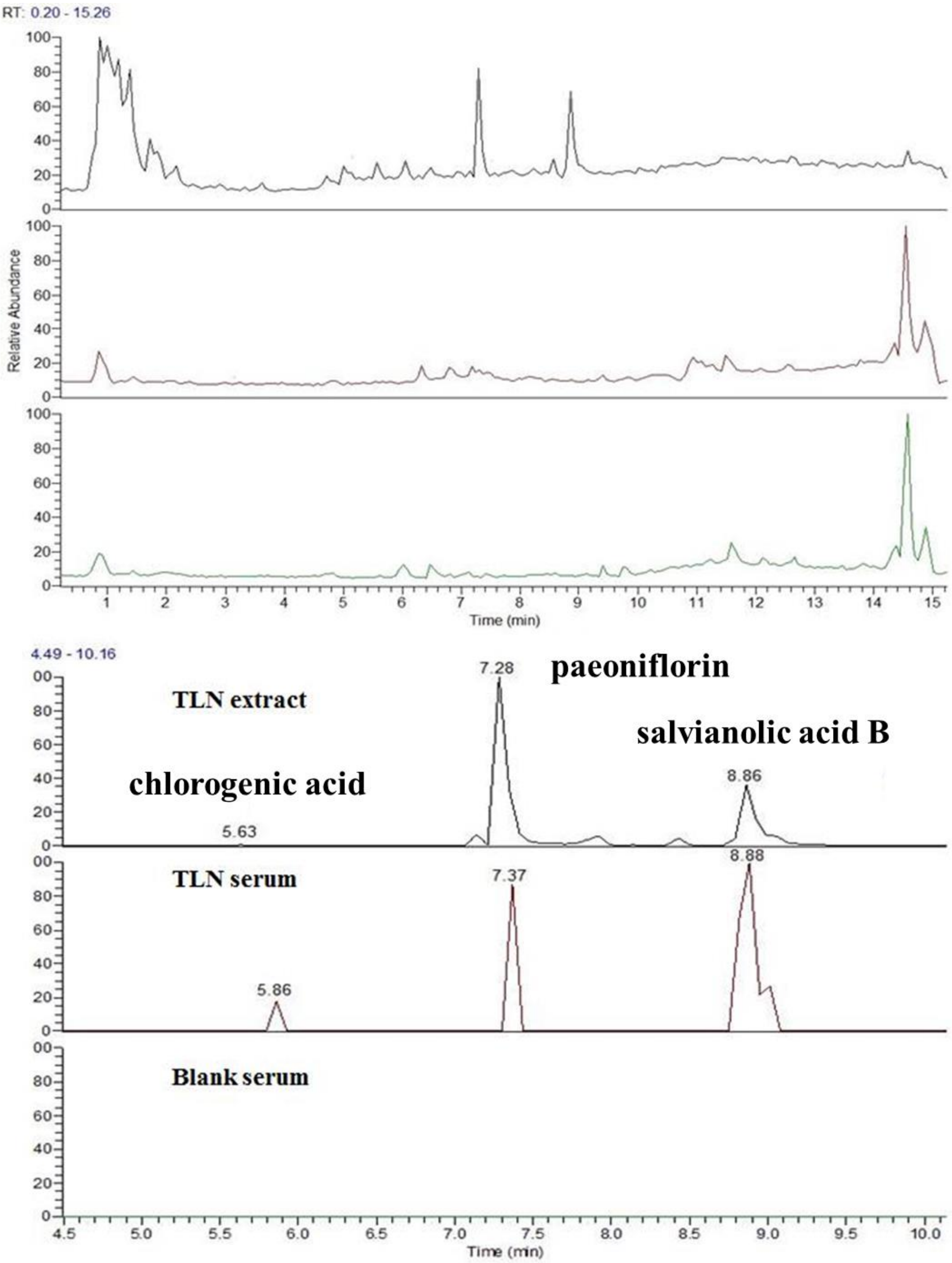

Fig. 1 HPLC-MS/MS total ion chromatogram and extract ion chromatogram in positive ion mode of TLN extract, TLN serum and blank serum.

Because GRP78 and CHOP are the key proteins of ER stress and Bcl-2 and Bax are CHOP downstream proteins, High content analysis focuses on location detection, and Western blot focuses on quantitative detection. In order to ensure the reliability of the conclusions, we used different methods to verify. But in order to avoid plagiarism, we have put these data in this

supplementary material.

1.2 TLN regulated related protein expression of ER stress-induced apoptotic pathway of RSC96 cells by high glucose

Up-regulation of GRP78 is known as sign of ER stress and CHOP is the most significant marker in ER stress-induced apoptosis. We first measured the two key proteins of ER stress-induced apoptosis and found that the expression of GRP78 in 150 mM glucose group increased significantly compared with the 25 mM glucose group ($P < 0.01$) (Fig.2C), it indicates that ER stress has occurred; the expression of CHOP in 150mM glucose group increased significantly ($P < 0.01$) (Fig.2D), it indicates that ER stress-induced apoptosis has occurred. TLN serum could decrease the expression of CHOP ($P < 0.05$, $P < 0.01$) (Fig.2D), then inhibited ER stress-induced apoptosis and relieved ER stress.

Studies showed that CHOP can inhibit the expression of Bcl-2. In this study, we found that the expression of Bcl-2 at 150 mM glucose group in different time points decreased with the increasing of CHOP expression (P<0.01) (Fig. 2D and E), TLN serum could also increase the expression of anti-apoptotic protein Bcl-2 ($P < 0.05$, $P < 0.01$) by inhibiting the expression of CHOP and decrease the expression of pro-apoptotic protein Bax ($P < 0.01$) (Fig. 2E and F), thus inhibiting ER stress-induced apoptosis

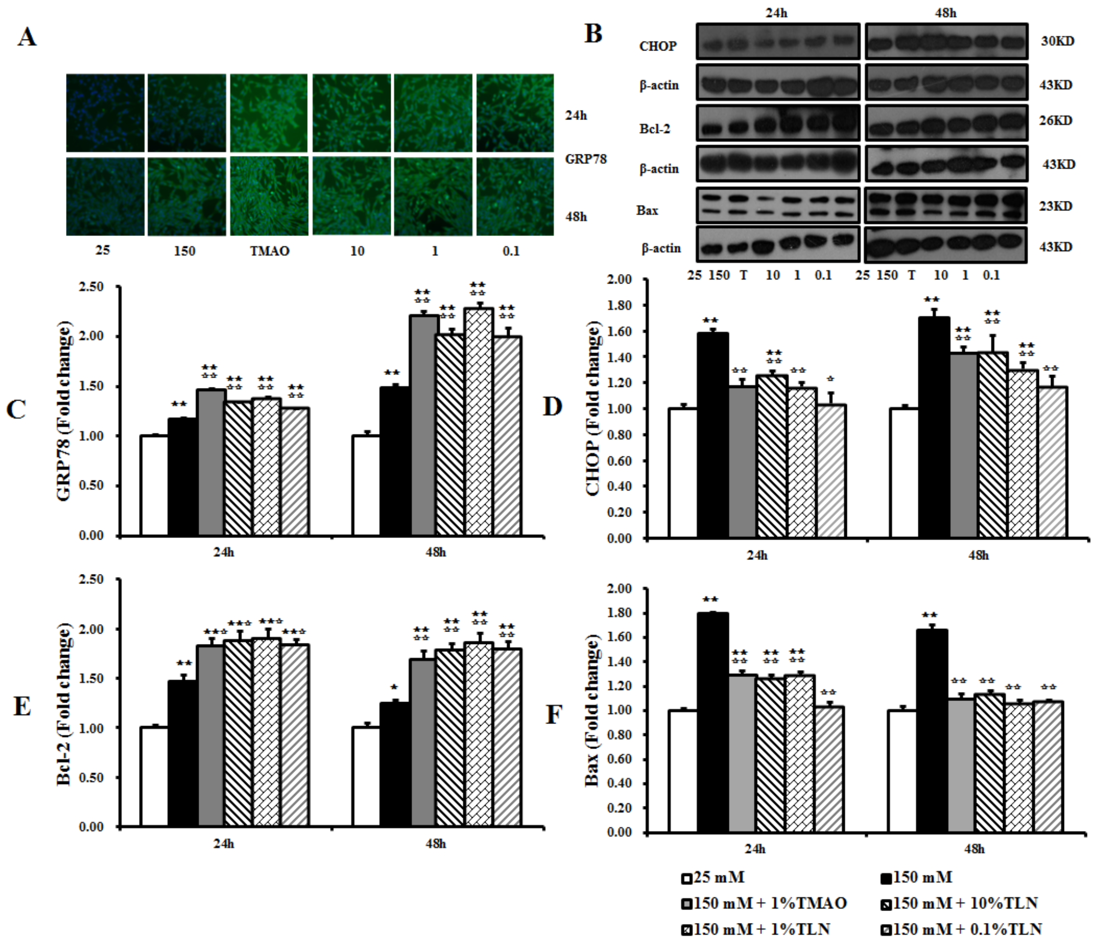

Fig.2 TLN increased the expression of GRP78 and Bcl-2 while decrease the expression of CHOP and Bax in high glucose-induced RSC96 cells A, Images of GRP78 relative protein level measured by high content analysis, images were viewed at a magnification of 10×; B, Images of CHOP, Bcl-2 and Bax relative protein level measured by western blot, normalized to β-actin; C-F, Summarized data of GRP78, CHOP, Bcl-2 and Bax, normalized as fold change of 25 mM glucose group. Data were analyzed by One-way ANOVA followed by least significant difference. Data were shown as mean ± SEM. n=4. ★★$P < 0.01$, ★$P < 0.05$, vs. 25 mM glucose; ☆☆$P < 0.01$, ☆$P < 0.05$ vs. 150 mM glucose; ••$P < 0.01$，•$P < 0.05$ vs. 150 mM glucose at 24 hours.
